# Supplementary material for: Pathophysiological Significance of Neutrophilic Transfer RNA-Derived Small RNAs in Asymptomatic Moyamoya Disease
Source: Cells. 2021 May 1;10(5):1086. doi: 10.3390/cells10051086 (PMC8147334; doi:10.3390/cells10051086)

| 2D Structure | Local AU | Position |
|---|---|---|

```
1645                           1675
                         8mer-1a
 5'-tcccATCCTTGGGCCTGGTAG-TCATGTGGGa-3' utr
        |||||  ::||  |  |||  :||||||||
 3'-uccuUAGGA--UUGG-CGAUCUGGUACACCCu-5' tRF
                    16151413      7 6 5 4 3 2
                   3' Pairing        Seed
```

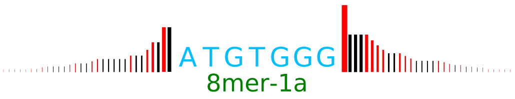


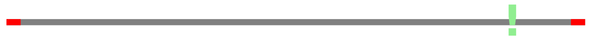

Supplement: Supplementary file 1 [file cells-10-01086-s001.zip › cells-1149161 3rd supplementary materials/Data Supplements/Data Supplement V.zip!/Data supplement 6-mRNAs targets of tsRNAs/tsRNA-Glu-TTC-010/Diagrams/tRF-Glu-TTC-010_vs_ENST00000354992(LARGE1).pdf]
